# Psychometric proprieties analyses of Psychological Vulnerability Scale for secondary school students

**DOI:** 10.3389/fpsyg.2024.1462830

**Published:** 2025-01-06

**Authors:** Odete Araújo, Otília Freitas, Gilberta Sousa, Isilda Ribeiro, José Carlos Carvalho, Silvana Martins

**Affiliations:** ^1^School of Nursing, University of Minho, Braga, Portugal; ^2^Health Sciences Research Unit: Nursing (UICISA: E), Nursing School of Coimbra (ESEnfC), Coimbra, Portugal; ^3^Nursing Research Centre, University of Minho, Braga, Portugal; ^4^Universidade da Madeira- Escola Superior de Saúde, Madeira, Portugal; ^5^CINTESIS@RISE_ Polo Madeira, Funchal, Portugal; ^6^Escola Superior de Enfermagem do Porto, Porto, Portugal; ^7^CINTESIS@RISE, Porto, Portugal; ^8^ProChild CoLAB Against Poverty and Social Exclusion Association, Guimarães, Portugal

**Keywords:** mental health, psychological vulnerability, secondary students, validation study, literacy

## Abstract

**Background:**

The concept of psychological vulnerability is associated with the individual’s maladaptive cognitive beliefs, such as self-criticism, perfectionism, and the need for external validation and approval, reducing the individual’s ability to cope with negative life experiences. This study aimed to explore psychometric proprieties of the Psychological Vulnerability Scale in secondary school students.

**Methods:**

A psychometric study was conducted with a non-probabilistic sample of 1,875 secondary school students (55.5% female) aged 15 to over 18 years. Participants completed a self-report questionnaire that included demographic information, the Psychological Vulnerability Scale, and a Positive Mental Health questionnaire.

**Results:**

Analysis revealed acceptable skewness values (between −0.557 and 0.6385) and kurtosis (ranging from −1.29 to −0.704). Confirmatory factor analysis (CFA) indicated excellent global fit indices, confirming the original structure. Invariance testing between genders demonstrated that the Psychological Vulnerability Scale was consistent for boys and girls (configural invariance) and that each item contributed similarly to the construct (metric invariance). The Psychological Vulnerability Scale showed good internal consistency, with an ordinal Cronbach’s alfa above 0.70. Reliability estimates, including inter-item reliability or MacDonald’s Omega, indicated mean item-inter correlations falling within the recommended range of 0.15–0.50.

**Conclusion:**

The Psychological Vulnerability Scale is a reliable tool that plays a crucial role in promoting the mental health of secondary school students by providing a structured way to assess their emotional and psychological state. They not only help in the early identification of signs of stress, anxiety, or depression but also facilitate the development of personalized interventions, fostering a continuously supportive and healthy school environment.

## 1 Introduction

Adolescence is a developmental phase marked by rapid and complex physical and mental changes, which occur during this life cycle stage (Orben et al., 2020). This transition from childhood to adulthood encompasses cognitive development and maturation, which are crucial for maintaining social relationships, managing problems and emotions, and shaping personality [World Health Organization (WHO), 2021]. How these physical and cognitive changes occur in young people will either positively or negatively impact their adult life. Therefore, greater or lesser psychological vulnerability among youngsters will be determined depending on how these changes are assimilated during adolescence [World Health Organization (WHO), 2021].

In Portugal, the report on The Health of Portuguese Adolescents, conducted in 2022, showed that despite most young people feeling happy (72.3%), more than 20% reported nervousness, bad mood (15.8%), sadness (11.6%), intense worry (22.8%), loss of control (26.4%), and approximately 20% reported an inability to deal with personal problems. Compared to previous national data (in 2018), there was a decrease in happiness and well-being levels and a worsening of physical and psychological symptoms, particularly fear, sadness, anxiety, and mood changes (Gaspar et al., 2022). National evidence on the vulnerability of young people aligns with global data, showing that approximately 14% of the adolescent population has mental health issues, according to WHO and the Agency for Healthcare Research and Quality [World Health Organization (WHO), 2021]. This evidence is more significant when considering that approximately 20% of mental health issues in young people start before the age of 14 (Scheiner et al., 2022). To understand the global challenges associated with assessing vulnerability during adolescence, it is imperative to conduct timely evaluations and identify vulnerable situations, thus preventing the worsening of mental health problems in young people.

The promotion of mental health throughout the life cycle, particularly during adolescence, has been widely advocated from a scientific perspective with clinical implications [World Health Organization (WHO), 2021]. In recent years, positive mental health has gained prominence through a salutogenic approach that is anticipatory and promotes mental health. This concept of positive mental health is widely discussed in various theoretical approaches. It focuses on resilience, emotional well-being, and psychological strengths rather than just the absence of mental illness. It emphasizes fostering a sense of purpose, autonomy, and social connectedness among individuals. This approach aims not only to prevent mental health issues but also to enhance overall well-being and quality of life across the lifespan, particularly during adolescence when developmental challenges can significantly impact mental health outcomes. Integrating positive mental health into clinical practice involves promoting coping strategies, enhancing self-esteem, and encouraging healthy lifestyle choices to build a foundation for lifelong mental resilience (Carvalho et al., 2022). The relationship between positive mental health and psychological vulnerability is complex. Positive mental health refers to states of emotional well-being, resilience, and coping skills, while psychological vulnerability involves susceptibility to stressors, trauma, and adverse conditions. Positive mental health can act as a protective factor against vulnerability. Individuals who cultivate a positive mindset and have access to emotional and social resources are better equipped to face adversities. Conversely, higher vulnerability can compromise mental health, creating a vicious cycle. Promoting positive mental health is crucial for mitigating psychological vulnerability and improving overall quality of life (Nobre et al., 2022).

Psychological vulnerability is defined as a set of cognitive schemas that increase sensitivity to stress and foster a sense of dependence on success or the approval of others. Individuals with psychological vulnerability may feel worthless when they experience failure or do not receive the approval of others (Sinclair and Wallston, 1999). Therefore, psychological vulnerability can result in dysfunctional or less adaptive patterns of thinking, feeling, and behavior (e.g., passivity, self-blame, social withdrawal, and catastrophizing), which may contribute to the development of psychopathology or a decline in psychological well-being (Sinclair and Wallston, 1999).

Psychological vulnerability has been positively associated with adverse psychological states, including anxiety, stress (Cox et al., 2001), self-alienation (Satici et al., 2013), social vulnerability (Sarıçam, 2015), and even heightened pain levels (Hansen et al., 2015). In contrast, it demonstrates negative correlations with key protective factors such as social competence, mindfulness, insight, resilience, social support, and self-efficacy (Akin, 2014; Gruebner et al., 2015; Kiamarsi and Abolghasemi, 2014; Satice et al., 2014), underscoring its critical role in shaping an individual’s psychological well-being and capacity to navigate stressors.

Due to its importance, psychological vulnerability is a variable that increasingly needs to be studied and measured using reliable instruments adapted to the population under study. The Psychological Vulnerability Scale (PVS) was developed by Sinclair and Wallston in the United States to identify predictors of vulnerability in adult populations with chronic illnesses. This six-item measurement instrument captures maladaptive cognitive dimensions or cognitions that enhance maladjusted reactions to stress, such as self-criticism, perfectionism, and the need for external validation and approval (Sinclair and Wallston, 1999; Sinclair and Wallston, 2010). According to Beck and Haigh (2014), psychological vulnerability encompasses dimensions associated with negative thinking perceived by individuals about life events, negative self-perceptions, negative perceptions of others, and rigid cognitive functioning. The original six-item measure version of the instrument developed by Sinclair and Wallston (1999) for adults with rheumatoid arthritis has demonstrated reliable and valid psychometric properties in both clinical and research contexts. The PVS has been translated and adapted for different contexts, including community and hospital contexts in the United States of America (Sinclair and Wallston, 2010), Scotland (Selbie et al., 2004) and Spain (Rueda et al., 2007). Globally, these translated versions respect the original version regarding the number of items, having only undergone cultural adaptations and adjustments for the population under study.

More recently, the PVS has been adapted for academic contexts to assess the psychological vulnerability of higher education students (Akin, 2014; Satice et al., 2014; Satici, 2016; Satici et al., 2015; Nogueira et al., 2017). Regarding the Portuguese context, the PVS was translated and culturally adapted by Nogueira et al. (2017). The authors conducted a study with 267 higher education students, confirming the PVS as a reliable instrument with adequate internal consistency and excellent stability over time. Cronbach’s alpha remained stable and adjusted to the original version. The construct validity of the Portuguese version of the PVS supported the original one-dimensional structure of six items, which aligns with previous literature (Sinclair and Wallston, 2010; Rueda et al., 2007; Satice et al., 2014; Akın et al., 2015; Selbie et al., 2004). The Portuguese version validated in higher education students has proven to be a valid, reliable, and suitable instrument for assessing the psychological vulnerability of higher education students. By adhering to the original version, the six-item scale is quick and easy to administer, and it is currently an efficient tool for supporting professionals in education and healthcare in assessing psychological vulnerability among higher education students. The same authors suggest that further studies are needed to provide more definitive evidence and test it in other groups of people so that this measurement instrument may gain more robustness and clinical utility. In addition, the PVS version adapted to the Portuguese context has been widely used in research involving higher education students. A previous study assessed the impact of recent years on the mental health of higher education students, focusing on their psychological vulnerability (Sequeira et al., 2022). The results revealed a negative impact on young people attending secondary education. Motivated by the significant mental health implications faced in recent years, specifically regarding the psychological vulnerability of secondary school students, this study aimed to explore the psychometric proprieties of the Psychological Vulnerability Scale for this demographic.

## 2 Methods

### 2.1 Procedures

This project is part of a multicenter study on mental health literacy among secondary school students. Students from the 10th, 11th, and 12th grades were recruited from two schools, one in the north of Portugal (Barcelos municipality) and the other in the autonomous region of Madeira. Before data collection, the research team provided detailed information by email to the schools’ directors, explaining the study objectives, data collection procedures, and the organization of the research teams. After study approval, the research team met with the class directors to address any questions regarding the study’s implementation and to obtain informed consent from students and their guardians. Also, before collecting the data, the research team ensured data collection harmonization and conducted instrument training. The study followed all ethical assumptions for human research. Before data collection in the classroom, students were provided with details about the nature and objectives of the study, anonymity and confidentiality, the duration of the questionnaire, and their right to withdraw from the study at any time. Written consent was obtained from students and their guardians. Data collection occurred between October 2022 and March 2023.

### 2.2 Ethics

The study was approved by the Ethics Committee of the Nursing School of Porto (reference number ADHOC_822/2020).

### 2.3 Measures

Table 1 presents all instruments used in this study. A Sociodemographic Questionnaire was completed by the participants including questions about age, gender, school year, and residence.

**Table 1:** Instruments description.

Instrument	Description	Item example
Sociodemographic questionnaire	A set of questions that allow the social and demographic characterization of the study sample.	Gender Age Parents’ educational level
Psychological Vulnerability Scale	Captures maladaptive cognitive dimensions or cognitions that enhance maladjusted reactions to stress, such as self-criticism, perfectionism, and the need for external validation and approval. Rated on a 5-point Likert scale.	2. I feel that I deserve better treatment than what I normally receive from others.
Positive Mental Health Questionnaire	Measures various aspects of positive mental health through six dimensions: personal satisfaction, pro-social attitude, self-control, autonomy, problem-solving/personal achievement, and interpersonal relationship skills. It includes 39 questions, rated on a 4-point Likert scale.	3. For me, it is difficult to listen to people’s problems.

The Psychological Vulnerability Scale was employed to gather information about psychological vulnerability. The PVS is a six-item scale ranging from 1-does not describe me at all to 5-describes me very well. Possible total scores range from 6 to 30, with higher scores indicating greater psychological vulnerability (Nogueira et al., 2017; Nogueira and Sequeira, 2024).

In addition, the Positive Mental Health Questionnaire (PMH) containing 39 questions on the way we think, feel, and act, was applied. The questions were grouped into six dimensions: personal satisfaction, pro-social attitude, self-control, autonomy, problem-solving and personal fulfillment, and interpersonal relationship skills. A previous study demonstrated that this questionnaire presented very good internal consistency for the global construct, with Cronbach’s alpha values for the dimensions ranging from 0.51 to 0.84, indicating good to very good reliability (Sequeira et al., 2014).

### 2.4 Data analysis

Data analysis was performed using IBM SPSS Statistics software (v.29, SPSS Inc., Chicago, IL) and JASP (v.0.18.3.0). Results were considered significant for *p* < 0.05. Participants with more than 10% missing data were excluded from the analysis. There were no missing values. A few moderate univariate outliers were identified by calculating the Mahalonobis distance (Tabachnick and Fidell, 2019) but were retained in the sample.

First, the psychometric sensitivity was assessed by examining measures of central tendency and distribution shape for the sample. Items with skewness above 3 and kurtosis above 7 (in absolute values) were rated as problematic (Kline, 2016).

Confirmatory factor analysis (CFA) was conducted to determine if the covariance structure of the model (Nogueira et al., 2017) matched the covariance structure of the data (Cheung and Rensvold, 2002). The global quality of factorial adjustment was assessed using several indices, such as chi-square (χ^2^), χ^2^ to degree of freedom ratio (χ^2^/df), comparative fit index (CFI), Tucker-Lewis index (TLI), and the root mean square error of approximation (RMSEA). Model fit was considered adequate for χ^2^/df values below 5, CFI and TLI of at least 0.90, and RMSEA below 0.08 (Brown, 2006).

The model was drawn using https://semdiag.psychstat.org (Mai et al., 2022). Factorial validity of the Psychological Vulnerability Scale was confirmed by ensuring that all items had standardized factorial weights higher than 0.50 (λij ≥ 0.50, λij2 ≥ 0.25) (Tabachnick and Fidell, 2019).

The measurement invariance of the PVS was tested through a sequence of restrictive models: testing for equal number of factors between male and female (configural invariance), ensuring equivalent factor loadings for each item (metric invariance), and restricting identical item intercepts (scalar invariance). Invariance was considered established when the added restrictions did not result in a significant deterioration of model fit. A non-significant χ^2^ difference test result and a Comparative Fit Index (ΔCFI) change value equal to or less than 0.01 supported measurement invariance testing (Byrne, 2010). Following conservative criteria of Chen (2007), measurement invariance was further confirmed when changes in CFI were less than 0.01 and changes in RMSEA were less than 0.015. Additionally, changes in SMRS were required to be less than 0.030 for metric invariance or 0.015 for scalar invariance.

Pearson correlations were performed to examine the relationship between scores on the Psychological Vulnerability Scale and the Positive Mental Health Questionnaire. Values above 0.80 indicated a very strong correlation, values between 0.60 and 0.80 revealed a strong correlation, values between 0.40 and 0.60 indicated a moderate correlation, values between 0.20 and 0.40 indicated a weak correlation, and values below 0.20 were considered negligible (Schober et al., 2018).

To assess internal consistency, Cronbach’s alpha, and McDonald’s Omega coefficients were computed for the PVS. Coefficients above 0.70 were considered acceptable, indicating good internal consistency (Ventura-León and Caycho-Rodriguez, 2017). Inter-item reliability was measured by computing the mean inter-item correlation for the Psychological Vulnerability Scale dimensions, aiming for values falling within the recommended range of 0.15 to 0.50 (Briggs and Cheek, 1986). Corrected item-total correlations were also calculated, with a cut point equal to or higher than 0.20 (Tabachnick and Fidell, 2019).

## 3 Results

### 3.1 Participants

The study included 1,875 adolescents (55.5% female) who completed a questionnaire through the Google platform. The inclusion criteria included adolescents with 15 years or more in secondary education. Age ranged from 15 years to over 18 years, with the majority being 17 years old (31.5%).

### 3.2 Descriptive and item analysis

Table 2 shows the descriptive statistics for items on the Psychological Vulnerability Scale. A five-point Likert-type scale was fully utilized in 100% of the items. Also, the average scores for items on the Psychological Vulnerability Scale ranged between 2.59 (SD = 1.41) for item 3 and 3.44 (SD = 1.29) for item 6, not distancing itself from the range of items median, as a central tendency, ranging from 2 to 4.

**Table 2:** Descriptive and item analysis.

Items	M	SD	Mdn	Minimum	Maximum	Sk	Ku
1. If I do not achieve my goals, I feel like a failure as a person.	3.27	1.32	3	1	5	−0.368	−0.984
2. I feel entitled to better treatment from others than I generally receive.	2.90	1.25	3	1	5	−0.079	−0.967
3. I am frequently aware of feeling inferior to other people.	2.59	1.41	3	1	5	0.272	−1.29
4. I need approval from others to feel good about myself.	2.23	1.30	2	1	5	0.638	−0.890
5. I tend to set goals too high and them become frustrated trying to reach them.	2.93	1.31	3	1	5	−0.005	−1.13
6. I often feel resentful when others take advantage of me.	3.44	1.29	4	1	5	−0.557	−0.704

All items presented adequate sensitivity, assuming absolute skewness and kurtosis values within the accepted limits of normal distribution (Kline, 2016). Finally, acceptable items’ skewness (ranging between −0.557 and 0.6385) and kurtosis (ranging between −1.29 and − 0.704) were identified.

### 3.3 Construct validity: confirmatory factor analysis, convergent, and discriminant validity

**Figure 1:**
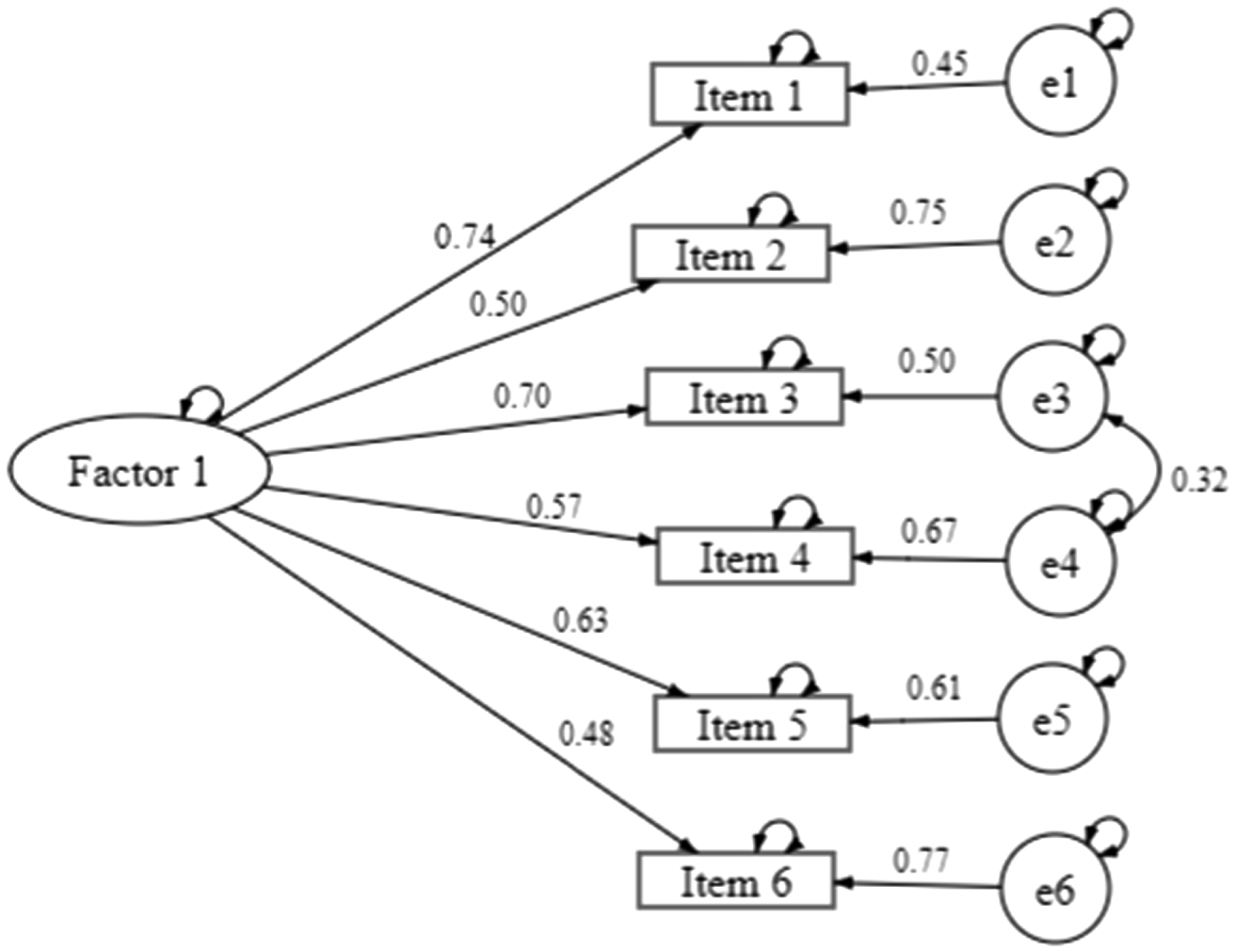
Model 2: factor loadings and covariances for the one first-order latent factors structure.

CFA fit indices for the two proposed models are presented in Table 3. Two models were evaluated. Model 1 representing the instrument with a one-factor structure, gathering all 6 items in a single dimension, following the original model (Sinclair and Wallston, 2010). Indicators of acceptable model fit were provided by chi-square statistic (χ^2^(9) = 132.970, *p* < 0.001, CFI = 0.956, TLI = 0.926, and RMSEA = 0.086, CI [0.073–0.099]). However, modification indices (considered as threshold 11) suggested a correlation between errors of items 3 and 4. The model modification indices were identified and the theoretical content shared between those items resulted in the improved solution of Model 2 (χ^2^(8) = 42.465, *p* < 0.001, CFI = 0.988, TLI = 0.977, and RMSEA = 0.048, CI [0.034–0.063]). The standardized factorial weights and individual item reliability for the model are presented in Figure 1.

**Table 3:** CFA models fit indices (*n* = 1,872).

Models	χ^2^	df	χ^2^/df	CFI	TLI	RMSEA
Model 1. One factor	132.970	9	14.8	0.956	9.926	0.926
Model 2. One factor with error correlation	42.465	8	5.31	0.988	0.977	0.048

RMSEA, Root mean square error approximation.

These results supported the one first-order latent structure (Model 2) for the original Psychological Vulnerability Scale (Nogueira et al., 2017). Furthermore, the quality of the Psychological Vulnerability Scale’s local adjustment was supported by factorial validity (λij ≥0.50, λij2 ≥ 0.25), considering that five items standardized factorial weights were higher than 0.50 (Tabachnick and Fidell, 2019), except for item 6 (λij2 = 0.23), with low saturation level, indicating that the latent dimension explained less than 25% of the result of this item.

### 3.4 Concurrent validity

The analysis of the correlations between the Psychological Vulnerability Scale and the Positive Mental Health Questionnaire (Table 4) indicated that psychological vulnerability was negatively correlated with the total score of positive mental health, personal satisfaction, pro-social attitude, autonomy, and interpersonal relationship skills. Conversely, positive correlations were found between psychological vulnerability and self-control, problem-solving, and personal fulfillment. All correlations were statistically significant and presented low to moderate magnitudes.

**Table 4:** Pearson correlations testing for concurrent validity between the Psychological Vulnerability Scale and the Positive Mental Health Questionnaire.

Items	1	2	3	4	5	6	7	8
1.Psychological vulnerability	--							
2.Positive mental health total score	−0.216^**^	--						
3.Personal satisfaction	−0.559^**^	0.539^**^	--					
4.Pro-social attitude	−0.092^**^	0.642^**^	0.144^**^	--				
5.Self-control	0.381^**^	0.423^**^	−0.243^**^	0.241^**^	--			
6.Autonomy	−0.559^**^	0.471^**^	0.609^**^	0.132^**^	−0.224^**^	--		
7.Problem-solving and personal fulfillment	0.301^**^	0.545^**^	−0.259^**^	0.458^**^	0.533^**^	−0.275^**^	--	
8.Interpersonal relationship skills	−0.124^**^	0.701^**^	0.347^**^	0.392^**^	0.201^**^	0.264^**^	0.258^**^	--

**p < 0.01.

The Positive Mental Health Questionnaire (QSM+, Portuguese version) used in this study was translated and adapted to the Portuguese population by Sequeira et al. (2014). This version provided evidence for reliability, content validity, and criterion validity in samples of the Portuguese adult population. Thus, during the psychometric evaluation of the QSM+ in the Portuguese population, the factorial structure proposed by Lluch (1999), Lluch (2002), and Luch-Canut (2003) underwent modifications, resulting in the final structure of the QSM+ (Portuguese version).

In the final structure of the QSM+, only the factors of personal satisfaction and self-control did not undergo modifications in the adaptation of the QSM+ to the Portuguese population. The version used in this study to define the positive mental health factors adheres to the QSM+ structure.

The items that constitute the QSM+ consist of statements reflecting ways of thinking, feeling, and acting that are common among individuals and relate to the six factors of positive mental health: personal satisfaction, pro-social attitude, self-control, autonomy, problem-solving, self-actualization, and interpersonal relationship skills (Sequeira et al., 2014; Lluch, 1999; Luch-Canut, 2003).

### 3.5 Multi-group CFA for measurement invariance across gender groups

Table 5 summarizes the fit indices for tests of measurement invariance across genders. According to Chen’s (2007) criteria, the results evidenced configural, metric, and scalar invariance between boys and girls. Specifically, there was a non-significant χ^2^ difference test result and ΔCFI <0.01, combined with ΔRMSEA <0.015 and SRMR <0.030 (for metric invariance) or < 0.015 (for scalar invariance).

**Table 5:** Results of Psychological Vulnerability Scale invariance tests.

Invariance tests	χ^2^ (df)	CFI	TLI	RMSEA (90%CI)	SRMR	Model comp	Δχ^2^ Δ(df)	ΔCFI	ΔRMSEA	ΔSRMR
M1: Configural invariance	43.827 (16)	0.989	0.979	0.043 (0.028–0.059)	0.019					
M2: Metric invariance	53.118 (21)	0.987	0.981	0.040 (0.027–0.054)	0.028	M1	9.291^n.s.^ (5)	−0.002	−0.003	0.018
M3. Invariance Scalar	67.883 (26)	0.985	0.982	0.039 (0.027–0.052)	0.031	M2	14.765 ^n.s.^(5)	−0.002	−0.001	0.003

*N = 1,873; Males: n = 833; Females; n = 1,040; χ2, chi-square goodness-of-fit statistic; CFI, Comparative Fit Index; TLI, Tucker-Lewis Index; RMSEA, Root Mean Square Error Approximation; Δ, Differences between two indices.*

### 3.6 Reliability of the Psychological Vulnerability Scale: internal consistency evidence

The Psychological Vulnerability Scale demonstrated good internal consistency, with a Cronbach’s alfa (*α*) of 0.78. Additional reliability estimates, including inter-item reliability and MacDonald’s Omega (*ω*), were provided to facilitate future comparisons with other studies.

Table 6 shows the internal consistency, mean inter-item correlations, and corrected item-total correlation range of the Psychological Vulnerability Scale, confirming the scale’s good internal consistency. Nevertheless, the mean inter-item correlations fell within the acceptable value range of 0.15–0.50 (Briggs and Cheek, 1986), and the corrected item-total also demonstrated good values above 0.20 (Tabachnick and Fidell, 2019).

**Table 6:** Internal consistency of the Psychological Vulnerability Scale.

Dimension	Cronbach’s Alfa	MacDonald’s Omega	Mean inter-item correlation	Corrected item-total correlation range
Psychological Vulnerability Scale	0.78	0.79	0.375	0.722–0.777

## 4 Discussion

This study aimed to explore the psychometric proprieties of the Psychological Vulnerability Scale (PVS) among secondary school students. Previous research utilizing exploratory factor analysis with a sample of young adults confirmed the scale’s robust psychometric characteristics, validating a one-factor solution (Nogueira and Sequeira, 2024). In this context, we conducted a confirmatory factor analysis (CFA) to further examine the scale structure. This step is crucial, as CFA allows testing the hypothesized factor structure and provides evidence of construct validity, which is essential when applying the scale to different populations.

Regarding the descriptive analysis of the items on the PVS, as previously described in the results section, the PVS is a five-point Likert scale. A global analysis of the items revealed that the mean scores of the items did not deviate from the median range of the items, with a central value varying from 2 to 4. Furthermore, all items showed adequate sensitivity, assuming absolute values of skewness and kurtosis within the accepted limits for normal distribution (Kline, 2016). Based on the analyzed data, acceptable skewness of the items (between −0.557 and 0.6385) and kurtosis (ranging from −1.29 to −0.704) were identified.

In the factor structure analysis, the procedures used the original model obtained from a previous study (Nogueira et al., 2017), confirming a one-factor structure through confirmatory factor analysis. The CFA results demonstrated very good global adjustment indices, confirming the previous structure. However, Item 6 “I often feel resentful when others take advantage of me” showed factor loadings below the recommended values, similar to findings from the exploratory factor analysis in the previous study (Nogueira et al., 2017). Considering the item content and this study sample, this result could be explained by the aspects related to adolescence. According to Piaget, egocentrism is a prominent feature of adolescent cognitive development, manifesting in various ways. Adolescents often exhibit a strong self-focus and tend to believe in the uniqueness and transformative power of their thoughts. They may develop elaborate “theories” or “systems” about themselves and the world, often in a somewhat naive manner. Additionally, adolescents begin formulating life plans, adopting adult roles, and expressing a desire for societal change. However, this heightened self-focus can lead to a relative inability to differentiate between their perspectives and societal norms, a phenomenon Piaget referred to as cognitive egocentrism. In their efforts to shape their environment according to their desires, adolescents may struggle to differentiate their constructs from broader societal expectations they seek to influence through these constructs. This cognitive egocentrism is commonly observed in adolescent writings, particularly in diaries and intimate confessions, where beliefs in the originality and potency of their ideas and their capacity to radically transform the world are often expressed. However, these expressions can sometimes be misinterpreted as signs of pathological messianism or megalomania (Galanaki, 2017).

Measurement invariance between genders was tested, and the results indicated that the basic organization of the PVS was supported for both boys and girls (configural invariance), with each item contributing similarly to the construct (metric invariance) (Byrne, 2010; Chen, 2007). This suggests that the PVS can be employed to compare vulnerability scores across different demographic segments. By analyzing latent mean scores and conducting group comparisons, it is possible to assess actual variations in vulnerability levels rather than differences in item interpretation (Putnick and Bornstein, 2016).

Understanding the factorial structure of the Psychological Vulnerability Scale in adolescents compared to other student groups is crucial for several reasons. Adolescents are at a unique developmental stage, characterized by specific psychological, emotional, and social challenges (Nunnally, 1978; Leite and Silva, 2019; Senna and Dessen, 2012; Vanderley et al., 2020). They often navigate issues such as identity formation, emotional regulation, and peer relationships, which differ from the developmental concerns faced by primary school or college students (Nunnally, 1978; Leite and Silva, 2019; Senna and Dessen, 2012; Vanderley et al., 2020). These age-related differences in concerns and challenges may influence how psychological vulnerability is experienced and perceived, potentially resulting in distinct factor structures across these groups.

Investigating whether the factorial structure differs between adolescents and other student groups is essential to ensure the validity and reliability of the scale across diverse populations. A consistent structure would suggest that the construct of psychological vulnerability is similarly understood across different stages of life. In contrast, differences in the factorial structure could indicate that the manifestation of psychological vulnerability varies with age, reflecting the unique stressors and coping mechanisms relevant to each group.

Furthermore, identifying potential structural differences has practical implications for developing targeted interventions. For instance, if adolescents emphasize factors like social approval and peer pressure, while college students focus on autonomy and self-efficacy, intervention strategies can be more effectively tailored to each group’s needs. This research contributes to a deeper understanding of psychological vulnerability, providing a foundation for future studies and interventions across various developmental, cultural, and contextual contexts.

Regarding the construct’s reliability, the Psychological Vulnerability Scale demonstrated good internal consistency, with an ordinal Cronbach’s alpha (Ventura-León and Caycho-Rodriguez, 2017; Nunnally, 1978) contributing to an overall sense of quality (Borsboom et al., 2004). Compared to a previous study (Nogueira et al., 2017), our data showed even higher reliability values. Other reliability estimates were presented, including inter-item reliability or MacDonald’s Omega, which were not previously estimated in the PVS previous study. MacDonald’s Omega indicated good internal consistency for the total sample, with mean item-inter correlations falling within the recommended range of 0.15–0.50 (Briggs and Cheek, 1986). The corrected item-total correlation range also demonstrated favorable results, exceeding 0.20 (Tabachnick and Fidell, 2019).

Overall, this study confirms the psychometric properties of the PVS for secondary school students and underscores the importance of understanding psychological vulnerability in this developmental stage. Given the increasing awareness of mental health issues among adolescents, tools like the PVS can be vital for educators, counselors and researchers in identifying students at risk and implementing timely interventions. Future research should focus on longitudinal studies to examine how psychological vulnerability evolves over time and its potential impact on academic and social outcomes.

The psychological vulnerability scale is a reliable instrument that enables health professionals to assess the psychological vulnerability of individuals in different clinical contexts (e.g., hospitals, health centers) and non-clinical contexts (e.g., schools, universities) throughout the life cycle (e.g., young people, adults, and older adults). Identifying psychological vulnerability allows for individualized, differentiated, and early interventions by health professionals. These study results also underscore the need for further research to refine the psychometric properties of the instrument. Future studies should include clinical and non-clinical samples to establish cut-off points, making the instrument more discriminative and sensitive. Improving the precision of the scale in assessing psychological vulnerability (e.g., low, medium, and high risk of psychological vulnerability) will facilitate more preventive and less remedial interventions, enabling anticipatory management of psychological vulnerability risk.

## 5 Conclusion

This study confirmed the strong psychometric properties of the Psychological Vulnerability Scale (PVS) among secondary school students, validating its one-factor structure through confirmatory factor analysis. The scale demonstrated good internal consistency and measurement invariance across genders, making it a reliable tool for assessing psychological vulnerability in adolescents. Given the growing importance of mental health awareness in this age group, the PVS offers valuable insights for educators and healthcare professionals in identifying at-risk individuals. Future research should focus on refining the scale through longitudinal studies and exploring its applicability in both clinical and non-clinical contexts, including establishing cut-off points to enhance its discriminative power. These advancements will further support preventive interventions and early identification of psychological vulnerability across diverse populations.

## Data Availability

The raw data supporting the conclusions of this article will be made available by the authors, after further request.
