# Erratum: Psychometric proprieties analyses of psychological vulnerability scale for secondary school students

**DOI:** 10.3389/fpsyg.2025.1601825

**Published:** 2025-04-09

**Authors:** Frontiers Production Office

**Affiliations:** Frontiers Media SA, Lausanne, Switzerland

**Keywords:** mental health, psychological vulnerability, secondary students, validation study, literacy

Due to a technical error, an earlier version of the manuscript was published instead of the latest, revised version.

The publisher apologizes for this mistake.

The original article has been updated.

